# Cytogenetic abnormalities and fragile-x syndrome in Autism Spectrum Disorder

**DOI:** 10.1186/1471-2350-6-3

**Published:** 2005-01-18

**Authors:** Kavita S Reddy

**Affiliations:** 1 Genzyme Genetics, Orange CA 92868, USA

## Abstract

**Background:**

Autism is a behavioral disorder with impaired social interaction, communication, and repetitive and stereotypic behaviors. About 5–10 % of individuals with autism have 'secondary' autism in which an environmental agent, chromosome abnormality, or single gene disorder can be identified. Ninety percent have idiopathic autism and a major gene has not yet been identified. We have assessed the incidence of chromosome abnormalities and Fragile X syndrome in a population of autistic patients referred to our laboratory.

**Methods:**

Data was analyzed from 433 patients with autistic traits tested using chromosome analysis and/or fluorescence in situ hybridization (FISH) and/or molecular testing for fragile X syndrome by Southern and PCR methods.

**Results:**

The median age was 4 years. Sex ratio was 4.5 males to 1 female [354:79]. A chromosome (cs) abnormality was found in 14/421 [3.33 %] cases. The aberrations were: 4/14 [28%] supernumerary markers; 4/14 [28%] deletions; 1/14 [7%] duplication; 3/14 [21%] inversions; 2/14 [14%] translocations. FISH was performed on 23 cases for reasons other than to characterize a previously identified cytogenetic abnormality. All 23 cases were negative.

Fragile-X testing by Southern blots and PCR analysis found 7/316 [2.2 %] with an abnormal result. The mutations detected were: a full mutation (fM) and abnormal methylation in 3 [43 %], mosaic mutations with partial methylation of variable clinical significance in 3 [43%] and a permutation carrier [14%].

The frequency of chromosome and fragile-X abnormalities appears to be within the range in reported surveys (cs 4.8-1.7%, FRAX 2–4%). Limitations of our retrospective study include paucity of behavioral diagnostic information, and a specific clinical criterion for testing.

**Conclusions:**

Twenty-eight percent of chromosome abnormalities detected in our study were subtle; therefore a high resolution cytogenetic study with a scrutiny of 15q11.2q13, 2q37 and Xp23.3 region should be standard practice when the indication is autism. The higher incidence of mosaic fragile-X mutations with partial methylation compared to FRAXA positive population [50% vs 15–40%] suggests that faint bands and variations in the Southern band pattern may occur in autistic patients.

## Background

In 1943 Kanner coined "infantile autism" (autism derived from Greek *autos*, or self) after observing 11 children, mostly boys, on the basis of their social isolation. Autism, often referred to as autistic disorder or infantile autism, is a complex behavioral disorder, which, by definition, develops prior to age three. Autism is defined completely on the basis of impairments in social interaction and communication, repetitive and stereotypic behaviors. Recent research has examined autistic traits in a population of twins and found that social impairment actually follows a unimodal distribution without a clear demarcation to separate cases of the disorder [1]. For this reason, future discussion of autism may be referred to as autism spectrum disorder (ASD) [2].

For most children, the onset of autism is gradual; however, about 30% have a "regressive" onset. Fifty to seventy percent of children with autism are defined as mentally retarded by nonverbal IQ testing. Seizures develop in about 25% of children with autism. About 25% of children who fit the diagnostic criteria for autism at age two or three years subsequently begin to talk and communicate, and by six or seven years blend to varying degrees into the regular school population. The remaining 75% continue to have a life-long disability requiring intensive parental, school, and societal support. There are no biologic markers, therefore the standard criteria, compiled by the American Psychiatric Association Manual of Psychiatric Diseases, 4th edition (DSM-IV), is the primary diagnostic reference used in the United States for autism. The causes of autism can be divided into "idiopathic," which comprises the majority of cases, and "secondary," in which an environmental agent, chromosome abnormality, or single gene disorder can be identified. About 5–10% of individuals with autism can be diagnosed with secondary autism; the remaining 90–95% have idiopathic autism. About 30% of children with idiopathic autism have complex autism, defined by the presence of dysmorphic features or microcephaly or a structural brain malformation [3]. About 70% of children with idiopathic autism have essential autism, defined as the absence of physical abnormalities.

A recent CDC case-finding study in Brick Township, New Jersey reported prevalence at 40 per 10,000 for autism [4]. While the latest epidemiologic study from the United Kingdom utilizing specialized visiting nurses who monitored child health and development at seven months, 18 to 24 months, and three years of age reported a prevalence rate of 16.8 per 10,000 for autism [5]. The sex ratio for autism has been estimated at 4 males:1 female [6,7].

Social cognition and communication in autism may be related to dysfunction in the amygdala, hippocampus, and related limbic and cortical structures. The cerebellum may also form part of a distributed neuronal network responsible for social cognition and communication. Serotonin is the neurotransmitter implicated in autism [8]

The single gene disorders in which secondary autism is observed include fragile X syndrome, tuberous sclerosis, phenylketonuria, Rett syndrome [3,9], Sotos, Neurofibromatosis I, Joubert syndrome [10], and Smith-Lemli-Opitz syndrome [11]. Risk to sibs of idiopathic cases is 75 times greater than the prevalence in the general population [7] and higher concordance for autism among monozygotic (60–90%) than dizygotic (0–10%) twins [12] argue for a genetic predisposition to idiopathic autism. Multiple independent whole genome scans and chromosomal abnormalities studies have pointed out several candidate regions on chromosomes 2q, 3q, 7q, 6, 13q, 15q, 16p, 17q and sex chromosomes. These regions possess candidate genes that have been screened for mutations or association with autism [13]. However, a clear involvement of a major susceptibility gene (or genes) in autism remains far from clear. The results from linkage studies and the drop in the concordance rates between monozygotic and dizygotic twins suggests that the genetic etiology of autism is certainly heterogeneous (different genes in different families), polygenic (more than one affected gene per individual) with epigenetic influences and allelic heterogeneity (different variants in the same gene may lead to different patterns of genetic disease) [13-15].

To gain further insight into secondary autism we have compiled data on patients with autistic traits tested for fragile-X syndrome using molecular methods and chromosome abnormalities using cytogenetic analysis and FISH.

## Methods

A search was initiated using the key word autism in the indication field of the Genzyme Genetics, Orange laboratory database. For each case the electronic data and/or files were reviewed. The referral center, age, sex, karyotype, fluorescence in situ hybridization (FISH) and fragile X results were extracted and tabulated.

G-banded metaphases were prepared using standard procedures and FISH was performed using the protocol provided by the manufacturer of the commercial probes (Vysis Inc., Illinois, MI).

Fragile X test: Isolated DNA was tested by both Southern blot analysis and Polymerase chain reaction (PCR) for the size and methylation status of the CGG repeat expansion within the FMR-1 gene. Southern blot analysis was performed with the probe StB12.3 on EcoR1 and Eag1 digested DNA. PCR products were separated by acrylamide gel electrophoresis and detected with a CGG repeat probe.

## Results

Patients with autistic traits were referred by physicians to our laboratory for genetic testing. The clinical diagnostic criteria applied were not specified on the test request forms.

A total of 433 patients with an indication of autism were sent to our laboratory for genetic diagnosis. The median age was 4 years. Sex ratio was 4.5 males to 1 female [354:79] (Table 1). A chromosome abnormality was found in 14/421 [3.33 %] cases. A Fragile-Xq27.3 was diagnosed in 7/316 [2.2 %].

**Figure 1:**
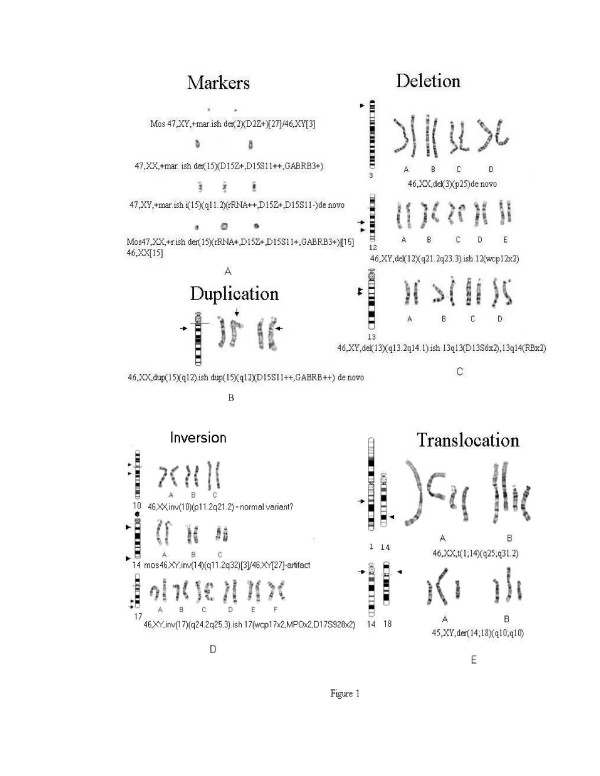
Chromosome abnormalities in patients with autistic traits (A) 4 markers: [multiple copies] derived from chromosome 2 [1], 15 [3] with nomenclature (B) 1 duplication of chromosome 15 with arrow denoting the region involved (C) 3 partial deletions (right homolog)(multiple copies) of 3p25, 12q21.2q23.3 & 13q13.2q14.1 with the ideogram, the arrows denote the deleted region. (D) 3 inversions (the right homolog) (multiple copies), inv(10)(p11.2q21.2), inv(14)(q11.2q32), inv(17)(q24.2q25.3) with arrows on the ideogram showing the inverted region (E) 2 translocations (partials in 2 copies, chromosomes involved (right) and their normal homolog (left)) one apparently balanced t(1;14)(q25;q31.2) and one unbalanced der(14;18)(q10;q10). The ideogram with arrows show the breakpoints

Chromosome abnormalities are summarized in Table 2: The aberrations were: 4/14 [28%] supernumerary markers from cs 15 [3] and cs 2 [1] (Fig 1a); 4/14 [28%] deletions of 2q37.3, 3p25, 12q21.2q23.3 and 13q13.2q14.1(Fig 1b); 1/14 [7%] duplication of 15q11.2q13; 3/14 [21%] inversions of 10p11.2q21.2, 17q23q25 (de novo) and 14q11.2q33 (mosaic) (Fig 1c); 2/14 [14%] translocations, one balanced t(1;14) and one unbalanced der(14;18) (Fig 1d).

The 3/4 marker/ring had a 15 centromere signal by FISH and had either nil, one or two signals for D15S11, that demarcates the involvement of the critical region of Prader Willi/Angelman syndrome (Table 2).

Fluorescence in situ hybridization was performed on 23 of 433 patients for reasons other than to characterize a cytogenetic abnormality, 3 to rule out Williams-Beuren Syndrome, 5 for DiGeorge syndrome, 7 for proximal duplication of D15S11, 6 for subtelomere rearrangements, 1-for subtelomere rearrangement & proximal duplication D15S11 and 1 for duplication D15S11, Smith-Magenis Syndome, and DiGeorge Syndrome. All 23 FISH tests were negative (Table 3).

**Figure 2:**
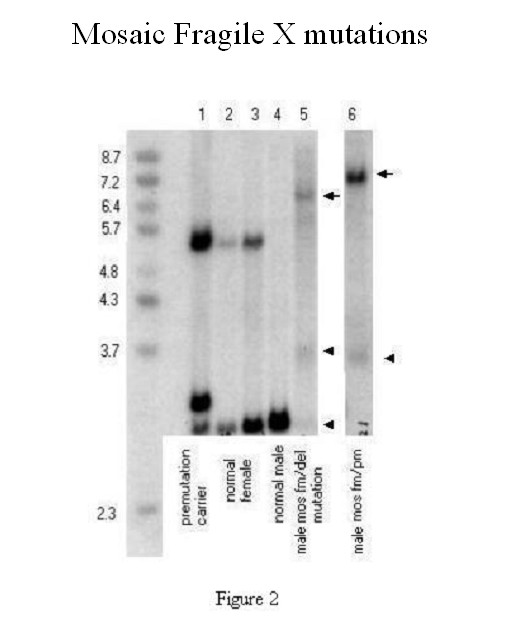
Mosaic fragile X mutations: Southern blot using probe StB12.3 on EcoR1 and Eag1 (methylation-sensitive enzyme) digested DNA. Lane 1 – permutation carrier, Lane 2 & 3 – normal female, Lane 4 – normal male, Lane 5 – an ASD male mosaic with fm (3.7 and 5.8–7.9 kb, 200–900 r)(arrows)/a deletion mutation (2.8 kb faint band, 30 r)(arrow). By PCR a 30 repeats and >200 repeats were amplified. Lane 6 – an ASD male mosaic fm (6.4 kb, 400 r)/pm (3.3 kb, 150 r).

Fragile X (Table 4): The mutations detected were: A full mutation (fM) and abnormal methylation in 3 [43 %] and mosaic mutations with partial methylation of variable clinical significance in 3 [43%]. The mosaic size mutations were: an fM [200–900 repeats(r)] / deletion mutation [30 r] with partial methylation. The faint bands for the full mutation and deletion mutation may be a reflection of the sample quality (Fig 2, Lane 5). Two size mosaics had permutation (pM) [150 r]/fM [400 r] (Fig 2), and pM [155 r]/fM [800 r] with normal and abnormal methylation. A premutation mosaic female carrier with an atypical EcoR1 and Eag1 pattern and a typical BssH1 pattern gave 2.8, 3.0, 5.2–5.4 Kb bands, the Eag1 pattern suggested a DNA sequence change [14%]. PCR gave reproducible bands corresponding to 29, 65, 80 repeats and a faint band for 39 repeats. Intermediate mutation (45–54 r) was found in 5 males and 2 females.

## Discussion

### Chromosomal causes of secondary Autism Spectrum Disorder (ASD)

About 1.7 % to 4.8 % of individuals with ASD have chromosome abnormalities, including unbalanced translocations, inversions, rings, and interstitial deletions and duplications (Table 5). The chromosome abnormalities that have been reported on more than one occasion are duplication of 15q, deletions of 18q, Xp, 2q and the sex chromosome aneuploidies 47,XYY and 45,X [16].

A recent FISH subtelomere study found one out of ten unselected patients with ASD had a subtelomeric 2qter deletion [17]. In our experience 7/7 ASD patients were negative for subtelomeric rearrangements.

Children with Down syndrome have autism more commonly than expected. The incidence was at least 7 % in one study [18]. This finding suggests that chromosome abnormalities may lower the threshold for the expression of autism.

In our study (4/14 = 28 %) and in other surveys, the common recurring chromosomal abnormality was duplication of the proximal 15q region (Table 5). About 1% of individuals with ASD have a chromosomal duplication in the Prader-Willi/Angelman region of proximal 15q [[19,20], present study 4/421[0.95%]]. The duplicated region q11.2q13 is on the maternal chromosome 15 in autistic patients [21-23]. Most commonly, this is a supernumerary isodicentric 15q chromosome detectable by routine cytogenetic studies or, less commonly, an interstitial duplication of the region detected by FISH analysis for the *SNRPN *gene region. These two chromosome abnormalities have only subtle effects on the physical phenotype. Supernumerary isodicentric 15q chromosomes are *de novo *occurrences. Duplication of proximal 15q may result from segregation of a parental chromosome translocation or an interstitial 15q duplication. An abnormal gene dosage within 15q11.2-q13 might cause susceptibility to autism.

**Figure 3:**
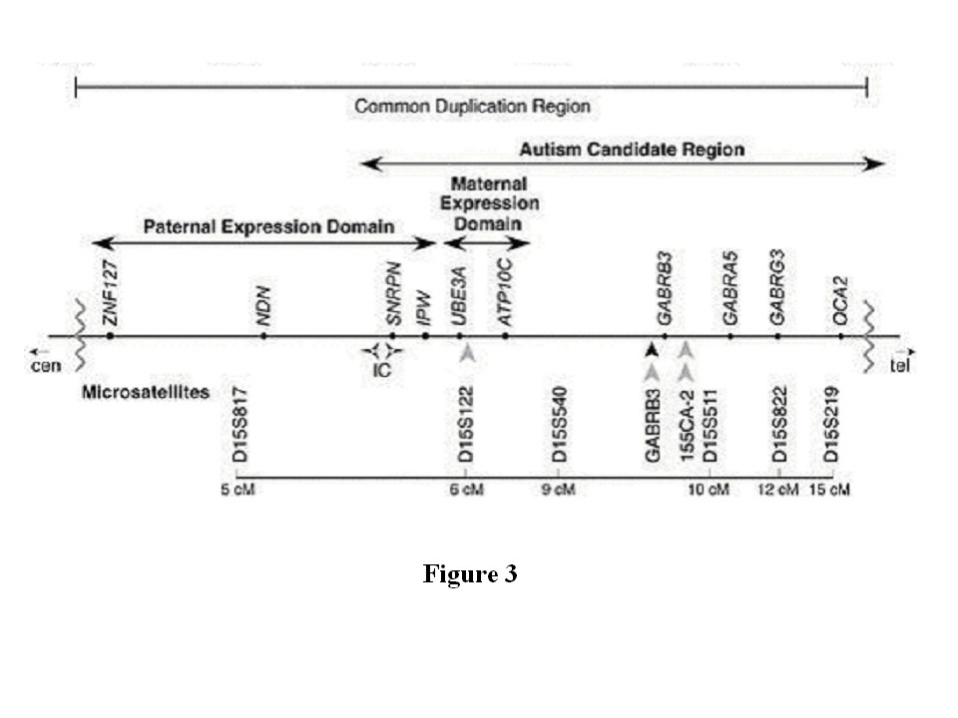
Chromosome 15q11-q13 region showing the autism candidate region. A schematic representation of the 15q11-q13 interval duplicated in autism cases and deleted in Prader-Willi/Angelman syndrome is shown. IC denotes the position of the 15q imprinting center. Loci corresponding to previous reports of linkage and association are indicated by dark and light arrowheads, respectively, below the map. (Adapted with permission from Sutcliffe J et al article "Dense linkage disequilibrium mapping in the 15q11-q13 maternal expression domain yields evidence for association in autism' in Molecular Psychiatry (2003) 8, 624–634)

**Figure 4:**
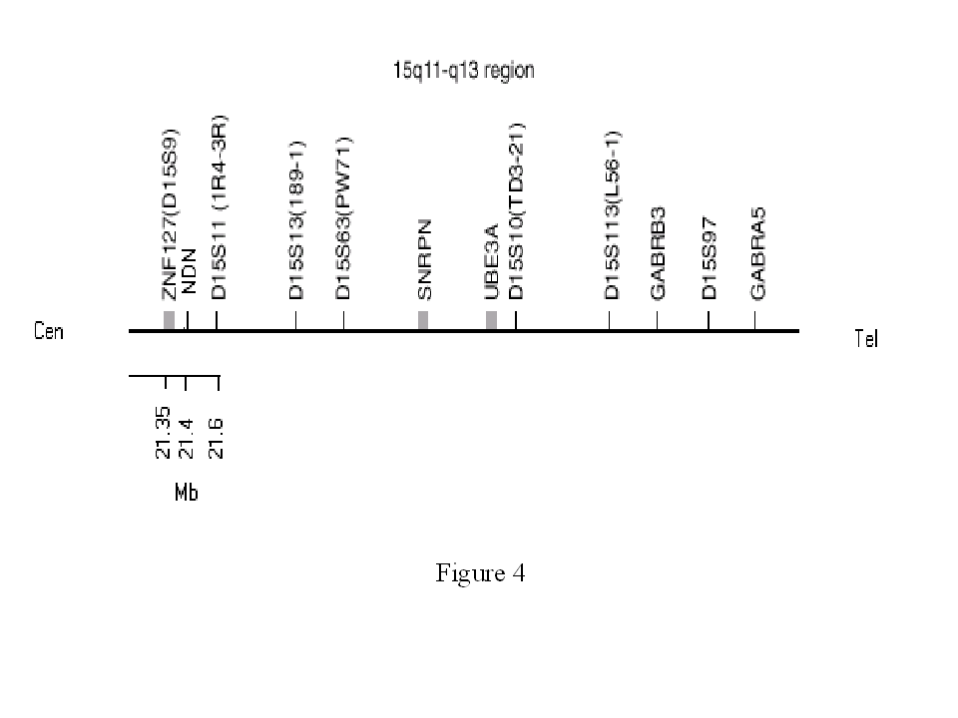
The map location of D15S11 probe used to characterize the markers

The 15q11-q13 region is shown schematically in Figures 3 &4. Chromosome 15q11.2-q13 has gained support as an autism candidate region on the basis of the association of maternally derived chromosomal duplications of this region with an autistic phenotype [20,24-27] and genetic evidence for linkage and allelic association in the same interval in chromosomally normal autism families [28-34]. The maternal specificity of chromosome 15 duplications in autism suggests a genomic imprinting effect. There are multiple imprinted genes in 15q11.2-q13, and two neurodevelopmental disorders exhibiting opposite patterns of genomic imprinting have been mapped to this region [35-37]. Interstitial deletion of 15q11.2-q13 specific for the paternal chromosome is the most frequent cause of Prader-Willi syndrome (PWS; MIM 176270), whereas maternal-specific deletion of the same common interval results in Angelman syndrome (AS; MIM 105830). The converse of Angelman Syndrome is observed in autism, that is a maternal duplication. The four causes of Angelman syndrome are 1) maternal deletion of 15q11.2q13, 2) paternal UPD15 3) mutations in UBE3A 4) mutations leading to imprinting errors of this region. A population-based study showed a high rate of ASD in AS [38]. But, a mutation was not identified in the UBE3A putative promoter or coding region in 10 idiopathic ASD patients [39]. Lack of expression of the maternally expressed UBE3A gene in the brain is thought to be the cause of AS. Since patients with deletions compared to other types of AS mutations have a more severe phenotype, suggests the involvement of additional gene losses, such as GABAA receptor gene cluster [40]. Transcripts increased in patients with duplications 15q11.2q13 are maternal UBE3A [41], maternal ATP10C [40,42] and other transcripts including antisense transcripts that could regulate gene expression [43] and may contribute to the duplication phenotype. Therefore, over expression of genes in 15q11-q13 probably confers ASD risk. Region proximal to D15S11 is considered to have no phenotypic effect. However, one of our patients had a marker 15 negative for D15S11, therefore, some duplications of 15, proximal to D15S11 and the autism candidate region may also influence susceptibility to autistic traits.

Initial studies to characterize the phenotype of 15q11.2q13 duplication patients have found variation among affected people including mental retardation, motor coordination problems, seizure disorder, and impairments in attention, communication, and social function (some but not all with ASD or attention deficit hyperactivity disorder (ADHD)) [44,45]. It appears there may be a parent-of-origin effect on the linkage and association signals in this region of UBE3A and ATP10C [46,32,33]. Further studies across data sets, and rigorous evaluation of potential functional effects of associated alleles, and a thorough assessment of haplotype transmission within ATP10C and neighboring genes would be conclusive. The majority of linkage and association data point to the *GABRB3 *gene, which is one of a cluster of γ-aminobutyric acid (GABA) receptor subunits that map to the distal, apparently nonimprinted segment of the duplicated region (Fig. 3). Although a number of groups have detected genetic effects at *GABRB3 *in independent autism populations [29-31], other studies have failed to replicate these observations [47-49].

Yardin et al 2002 [50] recommended a systematic screening by FISH, of chromosome region 15q11.2q13 in cases with autistic-like syndrome

A deletion of 2q37.3 was identified by FISH in a patient with autism and macrocephaly in our study. Wolff et al 2002 [51] also reported an autistic patient with a 2q37.3 deletion detected using subtelomere probes. Whole genome screens have suggested several chromosomal regions that are potentially associated with a susceptibility gene for autism. [52-57]. Three studies revealed positive linkage to 2q [52,53,57] and a third study demonstrated linkage to distal 2q in a subset of patients with autism and delayed onset of phrase speech [53]. Genomic scans are limited by the number of loci that are assessed; therefore, not all areas may be equally represented. It is important to note that telomeric regions may have increased meiotic recombination and may be under-represented in these types of analyses. Thus, the FISH approach is an important correlative study in the search for susceptibility genes. Macrocephaly in ASD: most children with autism are born with normal head circumference and about 20% meet the criteria for macrocephaly [58]. The increased rate of growth in head circumference appears to be most dramatic in the first year of life and corresponds to increased growth of the cerebral cortex as measured by MRI [59].

A de novo deletion of chromosome 3, del(3)(p25), was found in one case with ASD and development delay in our patient pool. A deletion of 3q region was found by Konstantareas & Homatidis 1999 [60]. Genome wide scan found a major susceptibility locus at 3q25-27 and there was also allelic association in families with autism spectrum disorder originating from a subisolate of Finland [61,62]. Animal models and linkage data from genome screens implicate the oxytocin receptor at 3p25-p26 [61,62].

To date, there have been no reports of 12q deletions in patients with ASD, to the best of our knowledge. For the first time we report an interstitial deletion 46,XY,del(12)(q21.2q23.3) in a patient with ASD, development delay, mental retardation, multiple congenital abnormality, and family history of Down syndrome.

46,XY,del(13)(q13.2q14.1)de novo was found in one of our patients' with ASD. Hyperserotonemia in autism is one of the longest-standing biochemical findings. The serotonin 2A receptor gene (HTR2A) on chromosome 13q14q21 is a primary candidate gene in autism. Converging data from recent genome screens also implicates the genomic region containing HTR2A [63-66]. Correlation of HTR2A disruption or deletion in our case with a 13q13.2q14.1 deletion and other 13q deletion in ASD patients would complement genome screen data.

The recent physical mapping of the serotonin 5-HT(7) receptor gene (HTR7) to 10q23 [67] raises the question if the inversion inv(10)(p11.2q21.2) present in our patient, which is considered a normal familial variant could have long range influence on HTR7 and susceptibility to autism in some cases.

So far there has been no observed association or link between chromosome 14 and ASD. Also, the mosaic inversion inv(14)(q11.2q33) [3/20] found in one of our patients is considered a cultural artifact when seen in 1or 2 cells.

In this study, a patient with inv(17)(q23q25)de novo, had ASD, hypotonia and developmental delay. Chromosome 17q shows association with autism by genome wide scans and in linkage studies [57,66,68]. There is also interest in 17q since serotonin transporter gene (SERT) has been mapped to17q11-q12 [69].

We report a patient with 18p deletion due to an unbalanced translocation between 14 and 18. Majority of the reported cases with autism involve deletion of 18q [70-72]. A deletion of the 18p-arm (at band 11.3) in about 50% cells and 50% of the cells with a duplication of the long arm in peripheral blood was described in a mildly obese girl with DSM-III-R autistic disorder and moderate mental retardation [73]. Another preschool girl with selective autism and a deletion of Chromosome 18p11.l has also been described [74]. She had communication problems consistent with a diagnosis of autism. However, in the area of reciprocal social interaction she was a little less deviant than most children and had no major behavior problems typical of autistic disorder. Linkage and association studies have suggested at least 2 candidate loci, one on the short and the other on the long arms of chromosome 18 [75].

One of the breakpoints in the balanced translocation, 46,XX,t(1;14)(q25;q31.2) was 1q25, in the present study. A recent report, links D1S1675 that maps to chromosome 1q24 with autism [76] using obsessive-compulsive behaviors as a restricting criterion for the analysis. The proximity of our breakpoint and D1S1675 may be coincidental or causal.

Although no cases with X- rearrangements were identified, in this study, the literature on abnormal X chromosome and autism is discussed as it has been cited in multiple cases. An autistic (ICD-10) woman had a translocation, t(X;8)(p22.13;q22.1) [77], a boy with "autistic disorder" had duplication of Xp22 [78], and a de novo Xp22.3 deletion was observed in 3 autistic females [79]. Further, mutations in cell adhesion genes NLGN4 on Xp22.3 and NLGN3 on Xq13 are reported in patients with autism [80]. Therefore, subtle Xp rearrangements have to be considered in the cytogenetic assessment of ASD patients

### Fragile X syndrome

The typical clinical picture in FRAXA includes mental retardation, macro-orchidism, large ears, and prominent jaw. Within neurons, the FMR protein (FMRP) interacts with mRNA and ribosomes, suggesting a role in regulating protein synthesis [81]. FMRP is heavily synthesized in dendritic spines in response to synaptic activity, and abnormal dendritic spine size and shape have been noted in FRAXA patients and fmr1 knockout mice [82]. These abnormalities may correspond to an abnormal postsynaptic response that weakens synaptic connections [83].

A multicenter study in Sweden [84] found fragile X in 13 of 83 boys (16%) with infantile autism but in none of 19 girls with infantile autism. Klauck et al. (1997) [85] concluded from molecular genetic studies of 141 patients from 105 simplex and 18 multiplex families that an association of autism with fragile X is nonexistent and that the Xq27.3 region is not a candidate for autism. Stoll (2001) [86] presented 11 children under the age of 8 years and the difficulties in diagnosis of fragile X syndrome at this age. The author concluded on the importance of fragile X DNA test for all children with mental retardation, autism, or significant developmental delay without a clear etiology. Whereas only a few percent of children with autism have fragile X syndrome, at least half of children with fragile X syndrome have autistic behaviors, including avoidance of eye contact, language delays, repetitive behaviors, sleep disturbances, tantrums, self-injurious behaviors, hyperactivity, impulsiveness, inattention, and sound sensitivities. The frequency of the fragile X syndrome among individuals with autism was ascertained up to 1993 using cytogenetic method. The incidence ranged from 12.7%–1.6%. However, these studies had different criteria for classifying positive cases. The differences were: the number of metaphases analyzed ranged from 20 to 100 and the cut-off range from 1% to 4% metaphases with a fragile X [84,87-94]. Using molecular analysis the incidence of fragile X syndrome was 5% (1/20) [95], 3.3%(1/30) [96], 12% (3/25) [97] and the present study 2.2 % (7/316). The difference in incidence between the present (2.2%) and previous studies (3.3% – 12%) may be due to small sample size in the other studies or clinical criteria for selection of patients or both.

#### Mosaicism for FRAX mutation: 

Forty-three percent of FRAX patients had a mosaic mutation in our study. The prevalence of males who carry a full mutation and a permutation is 15–20% [98-102] among the affected individuals. Nolin et al 1994 [103] analyzed a group of affected fragile X males by Southern blotting and found 41% (61/148) to be mosaic. This observation of 41% is significantly higher than previous reports 15–20%. The difference could be technical modifications, which permitted the identification of faint premutation bands in some patients. The higher percentage (43 %) of affected males with mosaicism in our study suggests that the occurrence of such individuals may be frequent in patients with ASD.

The degree of mental retardation seemed not to be influenced by the presence of premutation alleles in some of the cells and a full mutation in the rest of the cells [104]. While Merenstein et al 1996 [105] study suggests there may be some variation of clinical expression in fragile X males with a full mutation and permutation. It has been hypothesized that these mosaic cases should show higher levels of functioning than those who have only the inactive full mutation gene, but previous studies have provided negative or equivocal results. In one study, the cross-sectional development of communication, self-care, socialization, and motor skills was studied in 46 males with fragile X syndrome under age 20 years as a function of two variables: age and the presence or absence of mosaicism. The rate of adaptive skills development was 2–4 times greater in mosaic cases versus full mutation cases. FMR1 protein (FMRP) levels was shown to correlate with IQ, even in mosaic males for 38% of the IQ variance [106,107]. There was also a trend for cases with autism to be more prevalent in the full-mutation group [108]. However, we found a high incidence (43 %) of mosaic FRAX mutation in autistic patients, which requires confirmation in other FRAX studies of large cohorts of ASD patients.

#### Deletion mutation

The molecular mechanism of the FRAX is based on the expansion of a CGG repeat in the 5' UTR of the FMR1 gene in the majority of fragile X patients. The instability of this CGG repeats containing region is not restricted to the CGG repeat itself but expands to the flanking region as well. de Graaff et al 1996 [109] described four unrelated fragile X patients mosaic for both a full mutation and a small deletion in the CGG repeat containing region. Sequence analysis of the regions surrounding the deletions showed that both the (CGG)n repeat and some flanking sequences were missing in all four patients. The 5' breakpoints of the deletions were found to be located between 75–53 bp proximal to the CGG repeat. This suggests the presence of a hot spots for deletions in the CGG repeat region of the FMR1 gene and emphasizes the instability of this region in the presence of an expanded CGG repeat [110-113]. All reported cases had fragile X phenotype (which may be an ascertainment bias), and the deletion was usually a faint band suggesting a recent mutation in a small population of cells. Our case had an unusual Southern band pattern, consistent with the presence of both a full mutation (3.7 and 5.8–7.9 kb; 200–900 r)and a deleted (2.8 kb; 30 r) mutation with partial methylation. Since immunohistochemical staining or Western blot analysis was not performed to assess the FMRP production it precludes clinical correlation.

A 6-year old mosaic permutation female carrier had 29,65,80,39 repeats. The autism spectrum disorder in our patient may be due to diminished translational efficiency in FMRP production. Tassone et al (2000) [114] [have shown FMRP in 61% and 70% lymphocytes in two females 91/2 years and 33 years of age with 103/33, 180/30 repeats and IQ of 49 and 90, respectively. The first patient has physical, cognitive, and behavioral features of the fragile X phenotype but FMRP was in the normal range, while the second patient also had FMRP level in the normal range was treated for depression and has a history of ovarian cyst, premature menopause at 27 years, and a hysterectomy at 31 years. She also experienced social anxiety, panic attacks, mood swings, and mild obsessive compulsive behaviors. Johnston et al. (2001) [115] recently observed that emotional problems, including depression and interpersonal sensitivity, were more likely to occur in carrier females with >100 repeats. Austism spectrum disorder has been observed mostly in males with permutations [114,116,117]. The mosaic permutation in our female patient was 80 repeats as the largest expansion which is still below 100 repeats found in the affected cases in literature. Clinical correlation in our permutation mosaic case is hindered by the lack of FMRP studies and even so, the tissue distribution of mosaicism and FMRP profile would be the necessary phenotype determinant.

The significance of intermediate alleles found in 7/316 (Average age 4.36 years) of our patients requires further exploration on larger independent samples as they may raise the threshold for important developmental disabilities and/or physical features [116].

The limitations of our retrospective study were a lack of uniform clinical criterion for inclusion and limited behavioral diagnostic information for purposes of dissecting the phenotype. However, for autism diagnosis it may be reliable, as shown in a recent study [118] which examined the UK General Practitioner Research Database (GPRD) and found the diagnosis of autism among general practitioners in the UK had a high positive predictive value.

## Conclusions

In our experience, the incidence of chromosome (3.33%) and fragile-X (2.2%) abnormalities totals to 5.53% in a population of patients with an indication of autism sent for genetic testing. Since, 28% percent of chromosome abnormalities detected in our study were subtle; a high resolution cytogenetic study for ASD patients with a scrutiny of 15q11.2q13, 2q37 and Xp23.3 region should be standard practice. The higher incidence of mosaic fragile-X mutations with partial methylation in our ASD population vs incidence of mosaicism in reported populations with fragile X syndrome [50% vs 15–40%], suggests that faint bands and variations in the Southern band pattern may occur rather frequently in fragile X positive autistic patients. The mosaic FRAXA mutation with normal and abnormal or partial methylation may also enhance the threshold for autism spectrum disorder. Careful analysis and high quality gels are required to rule out the type of mutation in patients with autistic traits. FRMP estimates in positive cases would be an extremely useful adjunct especially in mosaic cases. Future studies are necessary to corroborate the high incidence of mosaicism and their role in ASD.

## Competing interests

The author(s) declare that they have no competing interests.

## Pre-publication history

The pre-publication history for this paper can be accessed here:



## Figures and Tables

**Table 1:** Demographics of the ASD patients tested

Demographics
Patients with ASD referred for genetic testing = 433
Median age = 4 years
Sex ratio = 4.5 males to 1 female

**Table 2:** The chromosome abnormality found in 3.33 % (14/421) patients with an indication of autism

**Sl #**	**Age**	**Sex**	**Clinical Indication**	**Chromosome Result**	**FISH Result**	**Fragile X Result**
				**Marker 4/14 = 28%**		

1	6.1	M	Autism	47,XY,+mar[27]/46,XY[3]	.ish der(2)(D2Z+)	NRM
2	5	F	Autism	47,XX,+mar	.ish der(15)(D15Z+,D15S11++, GABRB3+)	NT
3	3	M	Autism	47,XY,+mar de novo	.ish i(15)(q11.2)(rRNA++,D15Z+,D15S11-)	NT
4	5.1	F	Autism, MR	mos47,XY,+r[15]/46,XX[15]	.ish der(15)(rRNA+,D15Z+,D15S11+,GABRB3+).	NT

				**Duplication 1/14 = 7%**		

5	16.75	F	Autism	46,XX,dup(15)(q11.2q13)de novo	.ish dup(15)(q12)(D15S11++,GABRB3++)de novo	NT

				**Deletion 4/14 = 28%**		

6	6.5	F	Autism, DD, MR, macrocephaly		.ish del(2) (q37.3)(D2S447-)	NT
7	6.6	F	Autism, DD	46,XX,del(3)(p25) de novo		NT
8	11.7	M	Autism, DD, MR, multiple congenital abnormality, h/o DS	46,XY,del(12)(q21.2q23.3)	.ish 12(wcp12x2)	NRM
9	3.6	M	Autism	46,XY,del(13)(q13.2q14.1)de novo	.ish 13q13(D13S6x2),13q14(RBx2)	NT

				**Inversion 3/14 = 21%**		

10	2.8	F	Autism	46,XX,inv(10)(p11.2q21.2)*	NT	NRM
11	3.5	M	Autism	Mos46,XY,inv(14)(q11.2q33)[3]/46,XY[17]*	NT	NRM
12	3.25	M	Autism, hypotonia, DD	46,XY,add(17)(q23) or inv(17)(q23q25)de novo	.ish inv(17)(q24.2q25.3)(wcp17x2,MPOx2,D17S928x2)	NT
						

				**Translocation 2/14 = 14%**		

13	2.7	F	Autism	46,XX,t(1;14)(q25;q31.2)	NT	NT
14	3.3	M	Autism	46,XY,der(14;18)(q10;q10)	NT	NRM

DD = developmental delay, MR = mental retardation, DS = Down syndrome, NT = not tested NRM = normal *inv(10) is a normal familial variant and inv(14) is a frequently observed artifact of culture

**Table 3:** Summary of FISH cases

**Sl #**	**Age in years**	**Sex**	**Clinical Indication**	**Karyotype**	**FISH**	**FRAX**
1	5.25	M	Autism	46,XY	Normal ish 7q11.23(ELNX2)	
2	7	M	Autism, FTT, MR, DD	46,XY	Normal ish 7q11.23(ELNX2)	NRM
3		M	Autism	46,XY	Normal ish 7q11.23(ELNX2)	NRM
4	3.6	M	Autism, DD, hypocalcemia, cardiac defect, MR	46,XY	Normal ish 22q11.2(TUPLEx2)	
5	6.3	M	Autism, DD, hypotonia	46,XY	Normal ish 22q11.2(TUPLEx2)	
6	3.5	M	Autism	not ordered	Normal-ish 22q11.2(TUPLEx2)	
7		M	Autism	46,XY	Normal-ish 22q11.2(TUPLEx2)	
8		M	Autism	46,XY	Normal-ish 22q11.2(TUPLEx2)	NRM
9	6.5	M	Autism, DD, MR	46,XY	Normal, ish 15q12(D15S11x2)	NRM
10	11.6	M	Autism, DD	46,XY	Normal, ish 15q12(D15S11x2)	NRM
11	3.5	M	Autism	46,XY	Normal, ish 15q12(D15S11x2)	NRM
12	3	M	Autism	46,XY	Normal, ish 15q12(D15S11x2)	
13	2	M	Autism, DD,MR	46,XY	Normal-ish 15q12(D15S11x2)	NRM
14	2.5	F	Autism, DD,MR	46,XX	Normal ish 15q12(D15S11)	NRM
15	3.6	M	Autism, DD, patch hyperpigmentation	46,XY	Normal, ish 15q12(D15S11x2)	NRM
16	3.5	M	Autism, DD, seizures	46,XY	Normal- subtelomere panel	NRM
17	3.5	F	Autism, DD	46,XX	Normal-subtelomeres	NRM
18	4.6	M	Autism, DD	46,XY	Normal-subtelomeres	NRM
19	8.6	M	Autism, DD	46,XY	Normal-subtelomeres	NRM
20		M	Autism	46,XY	Normal-subtelomeres	NRM
21	2.7	M	Autism	46,XY	Normal-subtelomeres	ABN
22	16	M	Autism, DD, dysmorphic features, MR	46,XY	Normal, subtelomeres Normal, ish 15q12(D15S11x2)	
23	17.5	F	Autism	46,XX	Normal ish 15q11.2q13(D15S11x2), 17p11.2(SMSx2), 22q11.2(TUPLE1X2)	

M = male, F = female, NRM = normal, ABN = abnormal, DD = development delay, MR = mental retardation, FTT = failure to thrive.

**Table 4:** The details of Fragile X mutations found in 2.2 % (7/316) patients with a clinical indication of autism

**Sl #**	**Age In years**	**Sex**	**Clinical indication**	**FRAXA Result**	**Methylation**	**Chrom Result**	**FISH Result**
				**Full mutation 3/7= 43%**			

1	4.5	M	Autism	ABN-Full mutation (400,533,667repeats [r])	abnormal methylation	46,XY	NT
2	5	M	Autism	ABN-Full mutation (600–1100 r)	abnormal methylation	46,XY	NT
3	4.5	M	Autism, DD	ABN- Full mutation (267–933 r),	abnormal methylation	46,XY	NT

				**Mosaic mutation 3/7 = 43%**			

4	2.3	M	Autism	ABN-size mosaic Full mutation [800 r]/ premutation [155 r]	Normal & abnormal methylation	46,XY	NT
5	1.7	M	Autism	ABN-size mosaic Full mutation [400 r]/ premutation [150 r]	Normal & abnormal methylation	46,XY	Normal-sub tel
6	8.5	M	Autism, DD	ABN-mosaic Full mutation [200–900 r]/ deletion mutation [30 r],	partial methylation	46,XY	NT

				**Mosaic Premutation carrier 1/7 = 14%**			

7	6	F	Autism	ABN- premutation mosaic [29,65,80,39(light band)r]		46,XX	NT
	Av 4.36 years	2F, 5M		**Intermediate mutation **7 cases with 45–54r			

DD = developmental delay, NT = not tested

**Table 5:** Summary of reported population studies to assess the frequency of chromosome abnormalities (excluding fragile sites, polymorphisms and single cell abnormalities)

Study	Subjects karyotyped	Chromosome abnormality	Clinical diagnosis
Konstantareas & Homatidis 1999 [123]	127	6 [4.7%]	Diagnosed using the clinical criteria of autistic disorder by DSM-III (1983–1989)
		46,XY,inv(2)(p11q13)pat,3q+	
		47,XY,+mar	
		47,XY,+mar	
		47,XX,+13	
		47,XX,+inv dup(15)(pter→ q13::q13→ pter)	
		47,XY,+der(15)(pter→ q15::p11→ pter)de novo	
Gillberg & Wahlstrom 1985 [124]	46	2 [4.3%]	Diagnosed using the American Psychiatric Association (1980) criteria DSM-III
		47,XY,+21	
		47,XYY	
Lauritsen 1999 [122]	145	4 [2.8%]	Cases with psychotic symptoms before 2–3 years or beginning at 2–3 or later between 1969–1993
		47,XX,+mar,?t(13;22)	
		46,XX,t(9;10)(p23;q23.1)	
		46,XY,inv(10)(p11.21;q21.2)mat	
		46,XY,t(7;12)(q21.4;q15)de novo	
Li 1993 [121]	104	5 [4.8%]	Diagnosed using the American Psychiatric Association (1980 & 1987) guidelines, DSM-III & III-R
		47,XY,+21	
		46,XY/47,XY,+21 [12/88]	
		46,XY,t(5;6)(q13;p23)de novo	
		46,X,inv(Y)(p11q11)	
		46,fra(X)(q27.3),inv(Y)(p11q11)	
Weidmer-Mikhail 1998 [120]	59	1 [1.69 %]	DSM-III-R (1991–1995)
		Tetrasomy 15	
Ritvo 1990 [6]	233	9 [3.9%]	DSM-III (1984–1988)
		6-trisomy 21	
		Partial trisomy 8	
		Deletion 9p	
		46,XX,t(5q;11q)pat	
Wassink 2001 [119]	278	13 [4.7 %]	DSM-III,-III-R & -IV (1980–1999)
		46,XX,del(8)(p23)	MR, diaphragmatic hernia, hemivertebra, 2-vessel umbilicus, strabismus
		47,XX,der(14)t(14;?)(q22;?)	MR, abnormal facies & palate, failure to thrive
		46,XX,dup(15)(q11.2;q13)	MR, abnormal EEG, precocious puberty
		2–46,XY,del(15)(q11.2q13)	Mild MR Moderate MR & Seizures
		mat47,XX,+mar de novo 47,XX,+mar	McCune-Albright syndrome Microcephaly, abnormal facies
		47,XY,+del(15)(q22)	MR
		46,XY,del(16)(q13q22)	MR, Seizures, failure to thrive, abnormal facies, webbed neck macrocephaly, syndactyly
		46,XY,add(17)(q23)	MR & abnormal facies
		2–47,XX,+21	MR, heart murmur, esotropia, recurrent pneumonia. MR1VSD, pneumonia and seizures
		46,XY,add(22)(q13)	Macrocephaly and failure to thrive
Present	421	14 [3.3%]	Physician referrals to a genetic lab. 1995–2003 March
